# Management of Attention-Deficit Hyperactivity Disorder Children for Dental Procedures

**DOI:** 10.7759/cureus.36989

**Published:** 2023-04-01

**Authors:** Abdul Salam T.A, Manasila Ummer, Asem Abdullah Alowairdhi, Abdullah Khalid Alsubait, Sary Marwan Aljuhani, Abdulaziz Abdullah Alzahrani, Abdulmalik Ali Alqahtani

**Affiliations:** 1 Preventive Dental Science, College of Dentistry, King Saud bin Abdulaziz University for Health Sciences, King Abdullah International Medical Research Center, Ministry of National Guard Health Affairs, Riyadh, SAU; 2 Pedodontics and Preventive Dentistry, PSM College of Dental Science and Research, Kerala University of Health Sciences, Thrissur, IND

**Keywords:** attention-deficit hyperactivity disorder, fear, pharmacological management, behavior management, dental management

## Abstract

Background

A common psychiatric problem called attention-deficit hyperactivity disorder (ADHD) is characterized by impulsivity with resultant behavior issues and a very short attention span. The purpose of this study was to evaluate and compare the management of dental procedures in children with and without ADHD employing various behavior modification techniques.

Materials and methods

The study consisted of 121 children divided into two groups with 60 children diagnosed with ADHD and 60 children without ADHD between 7 and 15 years of age. Each of the three sessions, which were spaced a week apart, included a dental examination, oral prophylaxis, and a minor restorative procedure. The pulse rate (PR) and oxygen saturation (SpO_2_) were measured during each of these sessions. The study was conducted to evaluate the Tell-Show-Do (TSD) method, audiovisual distraction, and pharmacological management of children with and without ADHD during the dental procedure. IBM SPSS Statistics for Windows, Version 22 (Released 2013; IBM Corp., Armonk, New York, United States) was used to statistically analyze the findings. The mean values of the parameters from the three sessions were analyzed and compared using the Z-test.

Result

The children with ADHD included 39 (65%) boys and 21 (35%) girls, and the children without ADHD consisted of 27 (44.26%) boys and 33 (54.09%) girls. The mean values of the PR during sessions two and three were found to be statistically highly significant between the children with and without ADHD for TSD and audiovisual aids. In both groups, the mean SpO_2_ values for all the sessions were found to be statistically highly significant for the techniques evaluated (p<0.01). The change in the mean PR score for the ADHD children revealed a declining trend from sessions one through three for all the techniques evaluated (p<0.05), demonstrating a statistically significant effectiveness of the various techniques between the two groups and thereby revealing a decreased level of anxiety. Between sessions one and three, all of the three techniques showed a decreasing trend of SpO_2_ scores, with the exception of the pharmacological management of ADHD children (p<0.01) indicating that the uncontrollable ADHD children feel less anxious than in the other two approaches.

Conclusion

The findings of the study demonstrated that behavior management techniques were found to be effective at reducing anxiety in ADHD children than in children without ADHD. Our study further suggests that scheduling dental appointments into a series of short visits could enhance the effectiveness of the therapy and better cooperation of the children.

## Introduction

Attention-deficit hyperactivity disorder (ADHD) is characterized by impulsivity, hyperactivity, and inattention. ADHD is the most widespread neurobehavioral problem in children and one of the most common chronic illnesses affecting school-age children. It also affects every element of a child's life, including behavior, academic performance, and interpersonal connections with peers and family [1]. A thorough analysis of the literature revealed a global prevalence of between 7.8% and 9.5% for this disorder [2]. Boys are five times more likely to be affected than girls [3]. The Diagnostic and Statistical Manual of Mental Disorders-IV (DSM) recognizes three subtypes of ADHD: combined, primarily inattentive, and hyperactive/impulsive. DSM-IV categorizes ADHD into three subtypes: combined, mostly inattentive, and primarily hyperactive/impulsive [4]. Inattentive (restrictive) is a fourth subtype in the DSM-5 that can exist when the criteria for inattention are met, but only two symptoms of hyperactivity or impulsivity have persisted for the past six months [5]. Even though the exact cause of ADHD is unknown, studies indicate that the disorder may be caused by functional and structural disruption in the frontal cortex and basal ganglia regions of the cortico-basal ganglia-thalamo-cortical circuitry of the brain [6]. Studies on neurobiological variables show that genetics and neurochemistry are important influences [7]. 

Children with ADHD are more prone to have severe anxiety before receiving dental care [8]. The DMFT index revealed that they had a higher chance of experiencing carious lesions than the comparison group [9]. According to the data, children who used Dexedrine (dexamphetamine) or Ritalin (methylphenidate hydrochloride) or both medications had a higher prevalence of severe and unusual caries patterns [10,11]. These medications have the potential side effect of causing dry mouth, which may contribute to dental caries in children with ADHD [12]. Additionally, because of their hyperactivity, they are more likely to sustain traumatic tooth injuries, which could lead to worse dental conditions [13]. Regardless of age, people with ADHD can receive excellent dental treatment, even though symptoms typically persist into both adolescence and adulthood [14]. It is suggested that children with ADHD visit dentist offices more frequently to avert oral health issues [15].

The purpose of the present study was to evaluate and compare the management of dental procedures in children with and without ADHD employing various behavior modification techniques.

## Materials and methods

The study protocol was approved by the Institutional Ethics Committee of Yenepoya University with IRB number YUEC/140/3/12/12. Participants were chosen using a consecutive sampling technique from a clinical sample of children aged 7 to 15 years who had been sequentially sent to an outpatient list of a clinic for child and adolescent psychiatry. The estimation of the sample size was performed using the formula:

n = [DE*Np(1-p)]/ [(d2/Z21-α/2*(N-1)+p*(1-p)] where DE is the design effect assumed to be one, N is the finite population size, p represents the percentage frequency of the factor under study accounted as 7±5% based on a recent study report, and d is the marginal error (5%). The estimated minimum sample size was 101. After accounting for the potential dropouts, the final sample size of the study was increased to 121.

The study was carried out between May 3, 2021, and April 2, 2022 at Mangalore, using a control group of children from a government high school, who had not been diagnosed with ADHD or any other psychological issues according to the DSM-V criteria (American Psychiatry Association, 2013) [16]. Written informed consent was obtained from parents or legal guardians before the commencement of the study. The children who were interested in participating in the study were informed of its type and purpose. Anonymity was maintained at all levels of the study. Similar age and gender groups were considered for both the groups. Eight patients turned down the study invitation. During their initial visit to the psychiatry clinic, five patients agreed to participate in the study, but they chose not to visit the pediatric dentistry clinic.

Inclusion criteria for the study group

Children between 7 and 15 years old of both genders identified as having ADHD of DSM-V diagnostic criteria (diagnosis confirmation given by two independent psychiatrists on a standardized and structured interview) willing to provide consent either from parents or legal guardians were included in the study group.

Inclusion criteria for the control group

Children of both genders between the ages of 7 and 15 years without genetic or relevant mental illnesses willing to provide consent either from parents or legal guardians were included in the control group.

Exclusion criteria

Children with disorders of the central nervous system (CNS) such as epilepsy, serious injuries, and CNS infections co-existing with schizophrenia, bipolar affective disorder, any serious somatic disorders, chronic somatic diseases, persistent pharmacotherapy, and hormonotherapy were excluded. Children whose parents or local guardians were not willing were also excluded.

Study groups

The study consisted of 121 children with 60 children diagnosed with ADHD and 60 healthy children who have neither psychiatric diagnosis nor systemic illness. After psychological assessment, the children were referred to the dental college. There were three sessions of dental procedure, each scheduled a week apart. The first session consisted of dental examination and monitoring the pulse rate (PR) and saturation of oxygen (SpO_2_) using a pulse oximeter to evaluate the anxiety of the patient. During the second session, oral prophylaxis was performed, and from the previous visit, the cooperative attitude of the child was analyzed. The third session consisted of a restorative procedure which included a sealant placement. After each session, the child was given a stuffed toy or a stress-relieving smiley ball as a present for being cooperative during procedures and for following the instructions. The study was conducted to analyze the management of children with and without ADHD during a dental procedure by the tell-show-do (TSD) method, audiovisual distraction, and pharmacological management by examining the PR and SpO_2_.

The ADHD children were grouped into three with each consisting of 20 children. The normal children were categorized into three groups with 20 each in all groups. First, application of TSD was done. For this, a resident pedodontist demonstrated the mouth mirror as a mirror for teeth and how dental drill and suction work by using dental models and introduced an air-water syringe playfully as a water sprayer and as a small fan for teeth. The animations and pictures regarding dental caries were shown and explained as an information tool to help the child to decrease anxiety and be more acceptable. The child was given dental-like instruments to help the child to get familiar. Child-friendly language was used with euphemisms and terms appropriate to the developmental level of the children. 

Next was the audiovisual distraction, performed by showing the child’s favorite cartoons or animated videos on laptops using earphones. The videos included were a set of famous cartoons that are popular among their age groups. The behavior change was analyzed and recorded. The anxiety level was monitored based on the PR and SpO_2_ using a portable meter (Microtek Pulse Oximeter, Mumbai, India). Another type of management was the pharmacological behavior management. The dentist should be vigilant about the use of sedative drugs as these ADHD children on stimulant drugs may antagonize the sedative effect [17,18]. Before prescribing sedatives, consent from the child’s psychologist was obtained. Sedatives like Demerol, promethazine, and nitrous oxide were commonly used. The application of general anesthesia (GA) has not been considerably documented. Induction procedures were exceedingly difficult for children with ADHD undergoing elective surgery. According to a previous prospective study comparing the use of GA for children with and without ADHD, there was an increase in maladaptive behavior after the procedure [19].

Statistical analysis

The results were statistically analyzed by using IBM SPSS Statistics for Windows, Version 22 (Released 2013; IBM Corp., Armonk, New York, United States) software. The Shapiro-Wilks test was used to evaluate the normality of the distributions of parameters observed. The Z-test was applied for comparing the PR and SpO_2_ between the two groups. p< 0.05 value was considered statistically significant. The effectiveness of behavior management techniques was also compared by utilizing the difference in the mean score of PR and SpO_2_ from session one to session three.

## Results

The study sample comprised 121 children, out of which 60 (49.58%) children comprising 39 boys (65%) and 21 girls (35%) were with ADHD. On the other hand, 60 (50.41%) children were without ADHD encompassing 27 boys (45.90%) and 33 girls (54.09%). Mean values of the parameters obtained from the three sessions were examined and compared using the Z-test. Table 1 shows the results obtained from the TSD method.

**Table 1:** Impact of the tell-show-do technique among the study samples SD: Standard deviation; ADHD: attention-deficit hyperactivity disorder

Procedure sessions	PR (Mean±SD)			SpO_2 _(Mean±SD)		
	With ADHD (n=20)	Without ADHD (n=20)	p-value	With ADHD (n=20)	Without ADHD (n=20)	p-value
Session 1 (dental examination)	95.65±7.88	98.69±4.54	0.14	96.89±0.81	98.65±0.80	<0.001
Session 2 (oral prophylaxis)	92.45±6.74	99.12±3.12	<0.001	95.20±0.75	97.71±0.45	<0.001
Session 3 (restorative phase)	92.37±6.21	98.67±4.02	<0.001	94.90±0.79	96.90±0.69	<0.001

While the mean values of the PR during dental examination of session one were found to be insignificant between the groups (p>0.05), those of the PR during sessions two and three were found to be statistically highly significant between the children with and without ADHD (p<0.01). The mean SpO_2_ values were also found to be statistically highly significant in both the groups from sessions one through three (p<0.01). Table 2 reveals the results evaluated for audiovisual aids between the children with and without ADHD.

**Table 2:** Impact of audiovisual aids among the study samples SD: Standard deviation; ADHD: attention-deficit hyperactivity disorder

Procedure sessions	PR (Mean±SD)			SpO_2 _(Mean±SD)		
	With ADHD (n=20)	Without ADHD (n=20)	p-value	With ADHD (n=20)	Without ADHD (n=20)	p-value
Session 1(dental examination)	96.58±6.98	96.82±4.98	0.89	96.13±0.70	98.11±0.60	<0.001
Session 2 (oral prophylaxis)	91.54±6.40	97.28±4.72	0.002	95.84±0.96	97.80±0.74	<0.001
Session 3 (restorative phase)	90.60±6.91	97.45±4.97	0.001	95.46±0.69	97.72±0.58	<0.001

The difference in the PR between the two groups during session one was found to be statistically insignificant. Children with and without ADHD were found to have statistically highly significant PR values for sessions two and three indicating a difference in the anxiety level particularly in children with ADHD. The normal child without ADHD did not show an effective change in the PR as they were more on verbal control than the other group. In both the groups, the mean SpO_2_ values from sessions one through three were found to be statistically highly significant (p<0.01). Table 3 shows the findings of the pharmacological management of ADHD children.

**Table 3:** Impact of pharmacological management among the study samples SD: Standard deviation; ADHD: attention-deficit hyperactivity disorder

Procedure sessions	PR (Mean±SD)			SpO_2 _(Mean±SD)		
	With ADHD (n=20)	Without ADHD (n=20)	p-value	With ADHD (n=20)	Without ADHD (n=20)	p-value
Session 1 (dental examination)	98.65±7.10	97.21±4.11	0.44	98.49±0.78	97.01±0.88	<0.001
Session 2 (oral prophylaxis)	97.45±6.74	96.66±3.92	0.65	98.83±0.52	96.66±0.76	<0.001
Session 3 (restorative phase)	96.02±6.97	96.07±4.09	0.98	99.01±0.69	96.20±0.87	<0.001

It was determined that there was an insignificant difference in the PR values between the two groups across all three sessions. However, statistically highly significant differences in SpO_2_ scores were observed across the three sessions between the groups (p<0.01). The Z-test was used to compare the differences in mean values of all parameters between sessions utilizing various techniques, such as TSD, audiovisual aids, and pharmacological management (Table 4).

**Table 4:** Mean change in the parameters of PR and SpO2 from session one to session three using the various techniques among the study samples SD: Standard deviation; ADHD: attention-deficit hyperactivity disorder; PR: pulse rate; SPO_2_: oxygen saturation

	PR (Mean±SD)			SpO_2 _(Mean±SD)		
	Children with ADHD (n = 20)	Children without ADHD (n = 20)	p-value	Children with ADHD (n = 20)	Children without ADHD (n = 20)	p-value
TSD	3.28±1.67	0.02±0.52	< 0.001	1.99±0.02	1.75±0.11	< 0.001
Audiovisual aids	5.98±0.07	0.63±0.01	< 0.001	0.67±0.01	0.39±0.02	<0.001
Pharmacological management	2.63±0.13	1.14±0.02	<0.001	0.52±0.09	0.81±0.01	<0.001

The change in the mean PR score for the ADHD children decreased from session one to session three for all three techniques, demonstrating the effectiveness of the various techniques and revealing a decreased level of anxiety. The mean PR score for the children without ADHD varied from session one to session three, with a declining trend for TSD and pharmacological approach and an upward pattern for audiovisual aids. Between sessions one and three, all of the techniques used by the two groups showed a trend toward decreasing SpO_2_ scores, with the exception of the pharmacological management of ADHD children. Thus, it was evident from the increase in the SpO_2_ score that the pharmacological approach helped uncontrollable ADHD children feel less anxious than the other two approaches.

## Discussion

Due to their inability to keep attention, treating children with ADHD can be troublesome. The parents and their children experience increased anxiety while consulting a dentist which could be exhibited as overexcited behaviors in a child with ADHD. Many parents are concerned about how their child's behavior will affect other people, including dentists. It is usually beneficial to seek advice from the child's pediatrician or a medical specialist.

Additionally, it is best to schedule appointments in the mornings if the child is on stimulant medicine as these medication levels are at their highest. Furthermore, the children will be more attentive, less fatigued, and able to stay in a dental chair [20]. Atmella et al. have documented that children with ADHD might be more challenging to handle in the dental office due to the lack of establishing rapport, particularly with respect to teaching oral hygiene practices [21]. It is also advised to keep all instructions simple and to repeat them at various times. TSD was determined to be the most successful behavior management strategy using the Frankl behavior scale. TSD is helpful because it limits the unpredictable behavior of ADHD children, promotes cooperative behavior, and focuses their attention on the procedures. According to Charles, little tokens of appreciation may be offered throughout the procedure, and this could help to manage the child till the end of the treatment [22]. Short intermissions or recess during a lengthy dental procedure also serves as a crucial additional tool.

The PR and blood-SaO_2_ were the physiological measurements used in this investigation. According to several studies, stress and anxiety influence the heart rate and respiration, which affects the blood SaO2 [23,24]. Poiset et al. established measurement of SaO_2_ in the blood to be a valid approach to track dental anxiety [25]. The implication of the TSD method of behavior management was shown to have a considerable change in children with ADHD [26]. The TSD is the most frequently used method in pediatric dentistry because it is comforting for both the dentist and the child. The anxiety level of children in both the groups (with and without ADHD) in the present study was gradually decreased justifying the TSD as the most preferred means of patient education and behavior modification during the dental appointments as documented by Vishwakarma et al. [27]. TSD serves as a more reliable way of managing challenging children with ADHD in a dental office. Felicetti and Julliard documented TSD as the most effective behavior management method since it reduces the odd behavior of children having ADHD as well as exhibiting a cooperative nature along with distracting their attention on the dental procedures [28].

Children with ADHD are frequently frustrated because they are easily distracted due to their inattentive nature that may produce strange reactions which could interrupt treatment [29]. We employed an audiovisual method to divert their focus away from these disturbing inputs, thoughts, and feelings which was concurrent with the study conducted by Fakhruddin et al. [30]. The usage of audiovisual aid as a distraction tool in the present study produced statistically significant results with regard to heart rate variations during dental examinations. Similar to the findings of a study conducted by Fakhruddin et al., audiovisual distraction was found to be exceptionally helpful in the current study in lowering the patient's anxiety [31].

The practitioner must take into account the details of the additional regular medications prescribed for the children with ADHD to avoid drug interactions when planning for non-intravenous pharmacological (minimal or moderate) sedation. It makes sense to assume that a stimulant and sedative taken together would have a negative impact on one another. In a study conducted by Kerins et al., approximately 76% of dental professionals advised their patients to take the regular dose of ADHD medication before the consultation [32]. Successful dental treatment under pharmacologic minimal/moderate sedation is influenced by a variety of factors. The treatment location may have an impact on how the child interacts with their surroundings as well as the medicine regimen and dosage used (facility guidelines).

The outcome of a sedation appointment may also depend on the level of invasiveness of the planned dental procedure. The depth of sedation will be influenced by the pharmacologic differences between medication classes, but it is also important to consider the patient's inherent physiology. In our study, it was found that there is a slight change in SpO_2_ with the decrease of heart rate, and the children were found to be more attentive. Another similar study by Marshall et al. explained that the ADHD children who were given Midazolam for oral sedation showed little advantage [33]. The chances of idiosyncratic reactions were also documented in the literature. Thus, if managed properly, a general dentist or pedodontist can effectively treat children with ADHD in a dental setup.

It is advisable to give appointments in the morning so that the ADHD children will be more attentive and the effectiveness of medicine will be high, as it gets late its rebound effect may cause odd behavior [31]. The appointments arranged for children with ADHD should be made as short as possible to perform a quick dental procedure [32]. If a lengthy dental procedure is to be performed, GA would be more preferable after obtaining referral from the child’s physician [30]. Furthermore, giving frequent breaks will be more convenient for the child to engage in their own favorite activity. For both children with ADHD and without ADHD, long-term studies are required to evaluate the efficacy of behavior change techniques. Additionally, more research is warranted to identify the influence of different socioeconomic status on the changes in the behavior of ADHD children.

The dental surgeon and the pedodontists in particular should be trained to be at the frontline for the successful management of children with ADHD using appropriate behavior management approaches, rendering quick treatment with recurrent follow-up and for competent referrals to the pediatrician and neurologists if needed for the general wellbeing of the patient.

## Conclusions

Various behavior management techniques have been used. They all have some advantages and disadvantages and hence have to be utilized based on the patient's psychology. The findings of the study demonstrated that TSD, audiovisual aids, and pharmacological management were found to be effective at reducing anxiety in ADHD children than in children without ADHD.
